# Multidrug-resistant gonorrhea: A research and development roadmap to discover new medicines

**DOI:** 10.1371/journal.pmed.1002366

**Published:** 2017-07-26

**Authors:** Emilie Alirol, Teodora E. Wi, Manju Bala, Maria Luiza Bazzo, Xiang-Sheng Chen, Carolyn Deal, Jo-Anne R. Dillon, Ranmini Kularatne, Jutta Heim, Rob Hooft van Huijsduijnen, Edward W. Hook, Monica M. Lahra, David A. Lewis, Francis Ndowa, William M. Shafer, Liz Tayler, Kimberly Workowski, Magnus Unemo, Manica Balasegaram

**Affiliations:** 1 Global Antibiotics Research and Development Partnership (GARDP), Drugs for Neglected Diseases *initiative* (DND*i*), Geneva, Switzerland; 2 World Health Organization (WHO), Geneva, Switzerland; 3 Regional STD Teaching, Training & Research Centre, VMMC and Safdarjung Hospital, New Delhi, India; 4 Federal University of Santa Catarina, Florianópolis, Brazil; 5 National Center for STD Control, Chinese Academy of Medical Sciences & Peking Union Medical College Institute of Dermatology, Nanjing, China; 6 STD Branch, Division of Microbiology and Infectious Diseases, National Institute of Allergy and Infectious Diseases (NIAID), Rockville, Maryland, United States of America; 7 University of Saskatchewan, Saskatoon, Saskatchewan, Canada; 8 Centre for HIV & Sexually Transmitted Infections, National Institute for Communicable Diseases, Johannesburg, South Africa; 9 University of Alabama, Birmingham, Alabama, United States of America; 10 World Health Organization Collaborating Centre for Sexually Transmitted Diseases, South Eastern Area Laboratory Services, The Prince of Wales Hospital, Sydney, Australia; 11 Western Sydney Sexual Health Centre, Parramatta, NSW, Australia, and Marie Bashir Institute for Infectious Diseases and Biosecurity & Sydney Medical School-Westmead, University of Sydney, Westmead, Australia; 12 Skin & GU Medicine Clinic, Harare, Zimbabwe; 13 Department of Microbiology and Immunology, Emory University School of Medicine, Atlanta, Georgia, United States of America, and Laboratories of Bacterial Pathogenesis, VA Medical Center, Decatur, Georgia, United States of America; 14 Department of Medicine, Division of Infectious Diseases, Emory University, Atlanta, Georgia, United States of America; 15 World Health Organization Collaborating Centre for Gonorrhoea and other STIs, Örebro University, Örebro, Sweden

## Abstract

Emilie Alirol and colleagues discuss the development of new treatments for gonorrhea.

**Summary points**

- The number of gonorrhea cases is rising in many settings worldwide, and an increasing proportion of cases are multidrug-resistant. The choice of antimicrobials that can be used for treatment of gonorrhea is very limited, and resistance has even been reported to extended-spectrum cephalosporins, which are the mainstay of currently recommended antimicrobial therapy. Currently, only 3 new chemical entities are in different stages of clinical development for treatment of gonorrhea.
- In 2016, the Global Antibiotic Research and Development Partnership (GARDP) was launched by the World Health Organization (WHO) and Drugs for Neglected Disease *initiative*, which hosts and provides governance for GARDP.
- GARDP has worked together with experts from different regions to draft “ideal” and “acceptable” Target Product Profiles for the treatment of gonorrhea, reflecting medical need.
- Amongst other activities to combat antimicrobial resistance, GARDP has developed a plan to meet the urgent need for new drugs to treat gonorrhea.
- Over the next 7 years, this research and development proposal includes the following: exploring the introduction of a new clinical entity against gonorrhea; the identification of existing, suitable partner drugs; the recovery of previously abandoned, out-of-favor, and withdrawn antibiotics; and the development of simplified treatment guidelines for the empiric management of sexually transmitted infections.

## Gonorrhea: A growing, worldwide disease burden

Gonorrhea is among the most common sexually transmitted infections (STIs), with an estimated 78 million new cases in 2012 [1]. Countries with good surveillance have reported increases in cases of gonorrhea, such as an 11% rise between 2014 and 2015 in the United Kingdom [2], a doubling of cases among MSM (men who have sex with men) in France between 2013 and 2015 [3], a 5% rise between 2013 and 2015 in the United States [4], and an increase of 29%–146% in almost all Australian states between 2010 and 2014 [5], all reflecting longer-term trends. Decreasing condom use [6], increased urbanization and travel, poor infection detection rates, and inadequate or failed treatment [7] all contribute to this increase.

Gonorrhea affects high-, middle-, and low-income countries. The African region has the highest rates of gonococcal infections worldwide, with some 50 and 100 new infections per 1,000 women and men, respectively, every year [8]. In the US, it is the second most frequently reported notifiable infectious disease, accounting for 395,000 cases in 2015, a 13% increase from 2014 [4]; in Canada, a similar rise (15%) was reported.

Gonorrhea is a debilitating disease, which was responsible for an estimated 445,000 years lived with disability (YLD) in 2015 [9].

Urogenital gonorrhea may be asymptomatic in 40% of men [10] and manifests most commonly as urethritis [11]. It is also asymptomatic in more than half of women [12]. In men, untreated urethral infection can lead to epididymitis, reduced fertility, and urethral stricture. In women, when present, symptoms are nonspecific and include abnormal vaginal discharge, dysuria, lower abdominal discomfort, and dyspareunia. The lack of discernible symptoms [13] results in unrecognized and untreated infections, which can lead to serious complications. Overall, 10%–20% of female patients develop pelvic inflammatory disease (PID) and, consequently, are at risk for infertility [14]. Pregnancy complications associated with gonorrhea include chorioamnionitis, premature rupture of membranes, preterm birth, ectopic pregnancies, and spontaneous abortions [13,15,16]. Perinatal transmission occurs in 30%–40% of gonorrhea cases and occurs predominantly in low- and middle-income countries, where 3%–15% of mothers are infected. Infants of mothers with gonococcal infection can be infected at delivery, resulting in neonatal conjunctivitis (ophthalmia neonatorum). Such untreated conjunctivitis may lead to scarring and blindness.

Extra-genital infections are common in both sexes and frequently occur in the absence of urogenital infection [17,18]. Rectal infections are usually asymptomatic but can manifest as rectal and anal pain or discharge. Pharyngeal infections are mostly asymptomatic, but mild sore throat and pharyngitis may occur. Although bacterial concentrations are generally lower than in other infection sites, the pharynx is thought to be a favorable site for resistance emergence due to acquisition of resistance traits from commensal *Neisseria* spp. [19]. Disseminated gonococcal infections with gonococcal arthritis also occur. Because they are frequently asymptomatic, extra-genital infections often remain untreated, despite their key role in disease transmission.

Coinfection with other major STIs—HIV, Herpes Simplex Virus, *Chlamydia trachomatis*, *Mycoplasma genitalium*, and syphilis—are common and may result in synergistic effects on transmission and disease severity.

Almost all antibiotic classes used against gonorrhea have lost their efficacy because of resistance [20]. Sulfonamides, penicillins, early-generation cephalosporins, tetracyclines, macrolides, and fluoroquinolones can no longer be relied upon. The extended-spectrum cephalosporins (ESCs, i.e., cefixime and ceftriaxone), which represent the last remaining option for first-line empirical monotherapy, are also under threat, with resistance reported worldwide [7,21–24]. The WHO Gonococcal Antimicrobial Surveillance Programme (GASP) found that resistance is spreading especially in Asia, North America, Europe, Latin America and the Caribbean, and Australia, with large data gaps in Africa and Central Asia [25]. Reports of treatment failures with ESC are on the rise [26–38], and the first case of treatment failure with a dual therapy has recently been reported [7]. Fluoroquinolone, high-level azithromycin, and cephalosporin resistance have now been found in several countries [19,39–41].

*N*. *gonorrhoeae* displays extraordinary genetic versatility to achieve antimicrobial resistance (AMR), allowing horizontal gene transfer events with nonpathogenic *Neisseria* species that reside in different anatomical sites, particularly the pharynx [42–44].

The acquisition of multiple AMR traits, except perhaps for fluoroquinolone [45], does not appear to affect biological fitness, resulting in the persistence of strains that are multidrug-resistant (MDR) or extensively drug-resistant (XDR) even in the absence of antimicrobial selection pressure [45–47]. In the context of gonorrhea, MDR denotes resistance to current guideline treatments [48,49] including oral ESC, plus resistance to 2 or more of macrolides, fluoroquinolones, penicillins, tetracycline, aminoglycosides, and carbapenems. XDR denotes resistance to both oral and intramuscular ESCs or resistance to 1 type of ESC and spectinomycin, with resistance to 3 or more of macrolides, fluoroquinolones, penicillins, tetracycline, aminoglycosides, and carbapenems [48,49]. WHO recommends adapting treatment guidelines in areas with over 5% resistance.

Most patients are managed in the community, and because of limited diagnostic access and capabilities in many settings, gonorrhea treatment is empiric (i.e., symptom-based, without identification of the causative organism or definition of its antimicrobial susceptibility profile) and syndromic, in accordance with WHO guidance [50]. Treatment is based on the presence of easily recognized signs (e.g., urethral or vaginal discharge) and the provision of antibiotics that deal with the majority of, or the most serious, organisms responsible for the syndrome. With increasing resistance to ESC monotherapy, several countries have now adopted combination therapy with ESC plus azithromycin [51,52]. However, whether dual therapy actually delays resistance emergence is not supported by strong evidence [53], and strains resistant to either ESC or azithromycin are already in circulation [26–36,38]. In some regions of Africa and Latin America, less costly fluoroquinolones are still recommended, although they have been removed from WHO guidelines, and extensive resistance has been described [54–57].

Effective treatment of pharyngeal infections (regardless of resistance) is more difficult than treatment of urogenital infections; while the average cure rate for urogenital infection is 96%, rates drop to 79% and 84% (males and females) for oropharyngeal infections [58,59]. This may relate to insufficient drug exposure in the latter site. Worryingly, these infections most likely act as a reservoir, and persistence of pathogens at these sites jeopardize global efforts to slow the spread of resistant gonorrhea.

## Insufficient research and development for an urgent public health threat

As a disease that is not usually deadly but affects millions of people, gonorrhea control initiatives lack sufficient coordination and investment. With increasingly limited treatment options in the wider context of AMR, there is now growing concern that the threat of untreatable gonorrhea will become a reality. In February 2017, WHO listed *N*. *gonorrhoeae* among “High Priority” pathogens for research and development (R&D) of new antibiotics [60]. While hospital-acquired pathogens may have been highest on the list because of the high rates of mortality they cause, *N*. *gonorrhoeae* was notably included because infections are extremely widespread, cause substantial morbidity with a significant health cost for countries, can affect pregnant women and their babies, and develop AMR at a particularly rapid pace. Gonorrhea was also listed by the US Centers for Disease Control and Prevention (CDC) in the top “Urgent Threat” category of 18 drug-resistant threats to the US [61] and is included in similar AMR priority lists in the UK and Canada.

The current pipeline for gonorrhea treatments is severely depleted, with only 3 new chemical entities in various stages of clinical development. Two of these candidates are also being developed for other indications.

1. Solithromycin (Cempra Inc.) is an oral fluoroketolide with activity against gram-positive and fastidious gram-negative bacteria, including *N*. *gonorrhoeae*, *M*. *genitalium*, and *C*. *trachomatis* [62–64]. It targets 3 different prokaryotic ribosomal sites and showed good efficacy in a Phase II study [65], with a 100% efficacy for genital, oral, and rectal sites of infection in men and women. A Phase III trial is ongoing.
2. Zoliflodacin (Entasis Therapeutics) is a first-in-class spiropyrimidinetrione topoisomerase II inhibitor with activity against several pathogens, including *N*. *gonorrhoeae*, and *C*. *trachomatis* [66,67]. Zoliflodacin has been shown to be highly effective in vitro against a large collection of geographically and genetically diverse *N*. *gonorrhoeae* isolates [68]. Results from a Phase II trial showed high efficacy against urogenital infections (98%–100% microbiological cure rate, dependent on dose; clinicaltrials.gov NCT02257918). Over 90% of participants were male.
3. Gepotidacin (GlaxoSmithKline) is another bacterial topoisomerase II inhibitor, a novel triazaacenaphthylene with good in vitro activity against a wide range of drug-resistant bacteria, including MRSA (methicillin-resistant *Staphylococcus aureus*), ESBL (extended-spectrum β-lactamases)-producing *Enterobacteriaceae*, and *N*. *gonorrhoeae* [69]. A Phase II trial was recently completed, and 96.7% and 94.8% cure rates were achieved with doses of 1500 mg and 3000 mg, respectively (clinicaltrials.gov NCT02294682). As before, over 90% of the participants were male.

A global surveillance plan is outlined by Wi and colleagues in parallel with this R&D agenda [70].

The spread and incidence of gonococcal AMR is of great concern and has outpaced the development of new medicines, raising the prospect of untreatable gonorrhea [71,72]. A business-as-usual scenario will prevent achievement of the Global Health Sector STI Strategy's target, approved by the World Health Assembly in 2016, of a 90% reduction in the incidence of gonorrhea by 2030. The frequency of asymptomatic infections, rapidly changing antimicrobial susceptibility patterns, variety of AMR mechanisms, and, paradoxically, progress against HIV (resulting in a reduced use of condoms) make the control of AMR gonorrhea particularly challenging.

Commercial drug development for infectious diseases suffers from “market failure.” There are multiple reasons for this relating to how antibiotics are prescribed and sold, but also because stewardship initiatives may be diametrically at odds with the race against the “patent clock” to recoup drug development costs. Finally, there is competition from cheap generics, and the increasing need to combine antibiotics with other drugs, which brings formulation, costs, regulatory, and profitability challenges. Thus, there is an urgency to replenish the antibiotic drug discovery pipeline. In the shorter term, for gonorrhea, there is a need to advance, prioritize, and evaluate the 3 new molecules in the clinical pipeline, investigate new antimicrobial combinations, and reconsider the use of existing antibiotics. Moreover, for both new and existing drugs, there is a lack of clinical efficacy data on oropharyngeal infections.

The unmet treatment needs can be summarized as:

- No sustainable therapeutic option for MDR and XDR gonorrhea
- No evidence-based and sufficiently effective treatment for extra-genital infections, particularly oropharyngeal infections
- No evidence-based treatment for complications arising from initial urogenital infections

## An R&D proposal for gonorrhea

At the 68th World Health Assembly in 2015, WHO adopted the Global Action Plan on Antimicrobial Resistance. One of the Plan's initiatives was the launch of the Global Antibiotic Research and Development Partnership (GARDP; www.gardp.org) in May 2016 [73]. GARDP is hosted and governed by the Drugs for Neglected Diseases *initiative* (DND*i*) and has set up several programs aimed at developing new treatments in the short- to medium-term for STIs, neonatal sepsis, and an antimicrobial memory-recovery initiative. The latter aims to retrieve drugs and drug candidates (and associated expertise) whose use or development were halted in the past for reasons that no longer apply (e.g., Pharma portfolio considerations).

To better define the essential characteristics of new treatments for gonorrhea, and efficiently steer R&D activities, GARDP and WHO convened an international STI expert panel in mid-2016, who agreed on a Target Product Profile (TPP; Table 1). The requirements were split for short- and long-term targeted treatment, with each being further divided between “ideal” and “acceptable” profiles. Based on the needs identified above, and in line with the consensus TPP, GARDP has developed a comprehensive R&D strategy that is broken down into 4 complementary components.

**Table 1:** Consensus Target Product Profile (TPP) developed by the gonorrhea expert group.

	Short-term (up to 5 years)		Long-term (up to 10 years)	
	**Ideal**	**Acceptable**	**Ideal**	**Acceptable**
**Indication**	**(First-line) treatment of uncomplicated, urogenital gonorrhea (susceptible and MDR)**	**(First-line) treatment of uncomplicated, urogenital gonorrhea (susceptible and MDR)**	**(First-line) treatment of urogenital gonorrhea (susceptible and MDR, complicated and uncomplicated)**	**(First-line) treatment of urogenital gonorrhea (susceptible and MDR)**
	**First-line treatment of extra-genital gonorrhea (ano-rectal and oro-pharyngeal)**	** **	**First-line treatment of extra-genital gonorrhea (ano-rectal and oro-pharyngeal)**	
			**Treatment of *Chlamydia* infections**	
**Activity against coinfecting STI pathogens**	***Chlamydia trachomatis***		***Chlamydia trachomatis*, *Mycoplasma genitalium***	***Chlamydia trachomatis***
**Patient population**	**Adults and adolescents (aged 10–19 years)**		**Adults, children, and adolescents**	
**Clinical efficacy**	**97% (95% CI, 95–100)**	**95% (95% CI, 90–100)**	**97% (95% CI, 95–100)**	**95% (95% CI, 90–100)**
**Activity against ESC and macrolide-resistant NG strains**	**Yes**		**Yes**	
**Mechanism of action (target site, -cidal versus static; broad-spectrum versus narrow-spectrum)**	**Bactericidal/static**	**Bactericidal/static**	**Unique mechanism**	
	**Intracellular activity**	**–**	**Bactericidal/static**	**Bactericidal/static**
	**No cross-resistance**	**Limited cross-resistance**	**Intracellular activity**	**–**
	** **	** **	**No cross-resistance**	**Limited cross-resistance**
**Safety and tolerability**	**Well tolerated in pregnancy and lactation**	**–**	**Well tolerated in pregnancy and lactation**	**–**
	**No patient monitoring required post treatment**	**Minimal outpatient monitoring required post treatment**	**No patient monitoring required post treatment**	**Minimal outpatient monitoring required post treatment**
**Contra-indications**	**None**	**Pregnancy and lactation**	**None**	**Pregnancy and lactation**
**Drug–Drug Interaction profile**	**None**	**Minimal**	**None**	**Minimal**
**Route of Administration/formulation**	**Oral/IM, separated combination**		**Fixed-dose combination for orals**	**Copackaged loose combination**
**Dosing Schedule**	**Single dose**	**Multiple doses**	**Single dose**	**Multiple doses**
**Treatment duration**	** 1 day**	**Up to 5 days**	** Up to 3 days**	**Up to 5 days**
**Stability**	**Heat stable, 3-year shelf-life in climatic region IV**^**a**^	**Heat stable, 3-year shelf-life**	**Heat stable, 3-year shelf-life in climatic region IV**	**Heat stable, 3-year shelf-life**
**Cost (price/day of therapy)**	**Equivalent to current treatment regimens**		**Equivalent to current treatment regimens**	
**Time to patient availability**	**5 years**	**7 years**	**7 years**	**10 years**

ESC, extended-spectrum cephalosporin; IM, intramuscular injection; IV, intravenous injection; MDR, multidrug-resistant; NG, *Neisseria gonorrhoeae*; STI, sexually transmitted infection.

^***a***^Hot tropic/humid climate, simulated with 30°C and 65% relative humidity.

### Component 1: Accelerate the development of a new chemical entity

As part of this first component, GARDP will seek to accelerate development and registration of 1 new molecule for the treatment of uncomplicated gonorrhea and, in particular, to support the conduct of late-development activities (i.e., Phase III and IV trials). GARDP also aims to work with the patent holders to optimize the profile of the molecule along the lines of the TPPs.

To support access, stewardship, and conservation of the molecule, and keeping in mind the necessity to integrate it within existing guidelines, GARDP would then investigate possible combinations of the new molecule with existing antibiotics. In vitro studies would be initiated to investigate synergies, antagonisms, and activity against other STIs.

Finally, GARDP will seek to explore the clinical efficacy of the new therapeutic entity, alone or in combination, on (1) extra-genital gonorrhea and (2) patients coinfected with other STIs. This may entail investigating increased dosage or multiple-dose regimens, conducting additional pharmacokinetic/pharmacodynamic (PK/PD) investigations and gathering additional clinical data through subsequent trials in high-risk groups.

### Component 2: Evaluate the potential of existing antibiotics and their combinations

Several existing antibiotics have shown good antigonococcal properties in vitro and, for some, in patients: gentamicin, kanamycin, ertapenem, and fosfomycin. However, their efficacy remains to be confirmed in randomized clinical trials. Adequate PK/PD studies for these antibiotics are lacking, and more data are needed on their MIC (minimum inhibitory concentration) and the relationship between the MICs, PK/PD, and clinical outcomes. More data are also required for their utility in treating extra-genital and complicated infections. Other antibiotics have been abandoned but may deserve further investigation. The aminocyclitol spectinomycin was commercialized in the 1960s as a specific treatment for gonorrhea. Resistance rapidly emerged in some settings [74–76], and spectinomycin use was discontinued. But resistance is currently rare worldwide [20] and spectinomycin retains excellent activity against most gonococcal isolates. It is used in some European countries, China, and South Korea, but its availability in other regions is limited.

GARDP will aim to better understand the opportunities and liabilities of existing drugs and seek to identify optimal combinations through in vitro studies. Clinical efficacy of these combinations will be confirmed through trials involving groups with high STI burdens and sites in different countries that represent varied patterns of resistance.

### Component 3: Explore copackaging and development of fixed-dose combinations

Management of STIs often entails the coadministration of 2 or more antibiotics in order to cover all possible etiological agents. Copackaged products or fixed-dose combinations thus offer many practical advantages such as facilitating control over prescription, distribution, and administration of antibiotic combinations and reduce production costs. In addition, such combinations may increase compliance, and this may help to limit the emergence of AMR. Finally, fixed drug combination and/or copackaging offer a clear advantage in terms of stewardship. As part of this third component, GARDP will explore combinations and/or copackaging for the optimal combinations of new and/or existing antibiotics identified through the first 2 components.

### Component 4: Support the development of simplified treatment guidelines and foster conservation

To ensure the appropriate use of new treatments, GARDP and WHO will work with pharmaceutical companies, regulators, and other stakeholders to ensure that the newly developed antibiotics/combinations are globally accessible while at the same time used in an appropriate manner. The partners will support the development of evidence-based, regional/national treatment guidelines. This may entail carrying out observational studies and resistance surveys, in collaboration with WHO, to inform the integration of optimal combinations in STI guidelines. It may also involve observational studies to support the use of developed treatments in vulnerable populations (e.g., pregnant women and adolescents). GARDP will also work with partners to promote the appropriate use of new treatments by healthcare providers and patients by educating key stakeholders, supporting the conduct of pilot implementation studies, and monitoring of treatment use and emergence of resistance.

## Conclusions

The number of gonococcal infections is rapidly rising worldwide. Most worrisome, *N*. *gonorrhoeae* is an important member of the bacterial community that spreads AMR. Just 3 new clinical entities are in various stages of clinical development for treatment of gonorrhea today, in a therapeutic area that lacks a strong commercial interest. GARDP, a joint initiative founded by WHO and DND*i*, has begun to document ideal and acceptable profiles of antimicrobials for gonorrhea treatment. Four R&D routes have been outlined that require donor support: introduction of a new molecule for gonorrhea, identification of ideal combination partners among existing antibiotics, formulation of new fixed drug combinations, and establishment of a stewardship framework for the distribution and use of the new treatments. GARDP intends to work with its partners and other stakeholders to complete this roadmap and bring at least 1 new treatment into clinical practice by 2023.

## Abbreviations

AMR: antimicrobial resistance
DND*i*: Drugs for Neglected Diseases *initiative*
ESBL: extended-spectrum β-lactamase
ESC: extended-spectrum cephalosporin
GARDP: Global Antibiotic Research and Development Partnership
GASP: Gonococcal Antimicrobial Surveillance Programme
MDR: multidrug-resistant
MIC: minimum inhibitory concentration
MRSA: methicillin-resistant *Staphylococcus aureus*
MSM: men who have sex with men
PID: pelvic inflammatory disease
PK/PD: pharmacokinetic/pharmacodynamic
R&D: research and development
STI: sexually transmitted infection
TPP: Target Product Profile
XDR: extensively drug-resistant
YLD: years lived with disability
